# Deletion of Complement Factor H–Related Genes *CFHR1* and *CFHR3* Is Associated with Atypical Hemolytic Uremic Syndrome

**DOI:** 10.1371/journal.pgen.0030041

**Published:** 2007-03-16

**Authors:** Peter F Zipfel, Matthew Edey, Stefan Heinen, Mihály Józsi, Heiko Richter, Joachim Misselwitz, Bernd Hoppe, Danny Routledge, Lisa Strain, Anne E Hughes, Judith A Goodship, Christoph Licht, Timothy H. J Goodship, Christine Skerka

**Affiliations:** 1 Leibniz Institute for Natural Product Research and Infection Biology, Hans Knoell Institute, Jena, Germany; 3 Institute of Human Genetics, Newcastle University, Newcastle upon Tyne, United Kingdom; 2 Friedrich Schiller University, Jena, Germany; 4 Children's Hospital of the University of Cologne, Cologne, Germany; 5 Northern Molecular Genetics Service Laboratory, Newcastle upon Tyne Hospitals NHS Foundation Trust, Newcastle upon Tyne, United Kingdom; 6 Department of Medical Genetics, Queen's University, Belfast, United Kingdom; 7 Division of Nephrology, The Hospital for Sick Children, Toronto, Ontario, Canada; The Jackson Laboratory, United States of America

## Abstract

Atypical hemolytic uremic syndrome (aHUS) is associated with defective complement regulation. Disease-associated mutations have been described in the genes encoding the complement regulators complement factor H, membrane cofactor protein, factor B, and factor I. In this study, we show in two independent cohorts of aHUS patients that deletion of two closely related genes, complement factor H–related 1 *(CFHR1)* and complement factor H–related 3 *(CFHR3),* increases the risk of aHUS. Amplification analysis and sequencing of genomic DNA of three affected individuals revealed a chromosomal deletion of ∼84 kb in the RCA gene cluster, resulting in loss of the genes coding for CFHR1 and CFHR3, but leaving the genomic structure of factor H intact. The *CFHR1* and *CFHR3* genes are flanked by long homologous repeats with long interspersed nuclear elements (retrotransposons) and we suggest that nonallelic homologous recombination between these repeats results in the loss of the two genes. Impaired protection of erythrocytes from complement activation is observed in the serum of aHUS patients deficient in CFHR1 and CFHR3, thus suggesting a regulatory role for CFHR1 and CFHR3 in complement activation. The identification of *CFHR1/CFHR3* deficiency in aHUS patients may lead to the design of new diagnostic approaches, such as enhanced testing for these genes.

## Author Summary

Hemolytic uremic syndrome (HUS) is a severe kidney disease, which is characterized by hemolytic anemia, thrombocytopenia, and acute renal failure. The nondiarrhea-associated form, also known as atypical HUS (aHUS), is rare, sometimes familial, often recurrent, and has a poor outcome. Several studies have shown that aHUS is associated with mutations in genes coding for complement regulators, which leads to defective regulation of complement activation, particularly at cell surfaces. We report a novel susceptibility factor for aHUS in the form of a chromosomal deletion of a large (**∼**84 kb) genomic fragment in the regulators of complement activation gene cluster at Chromosome 1q32. This deletion is a result of nonallelic homologous recombination and leads to the loss of two genes, *CFHR1* and *CFHR3,* which encode factor H–related proteins 1 and 3, respectively. We recommend diagnostic screening of aHUS patients for these susceptibility factors.

## Introduction

Atypical hemolytic uremic syndrome (aHUS) is characterized by a triad consisting of microangiopathic hemolytic anemia, thrombocytopenia, and acute renal failure in the absence of a preceding diarrheal illness. aHUS can be either sporadic or familial. Defective complement regulation occurs in both sporadic and familial aHUS. Disease-associated mutations have been described for the genes encoding the complement regulators complement factor H (CFH), membrane cofactor protein, factor I, and factor B [1–4]. In addition, autoantibodies to factor H have been reported in aHUS patients [5]. Recently, we showed in a family with aHUS that nonallelic homologous recombination [6] results in the formation of a hybrid gene derived from exons 1–21 of *CFH* and exons 5–6 of complement factor H–related 1 *(CFHR1)* [7]. The protein product of this hybrid gene is identical to the aHUS-associated CFH mutant S1191L/V1197A, which arises through gene conversion [8]. *CFH* and the genes encoding the five complement factor H–related proteins *(CFHR1–CFHR5)* reside in a centromeric 355-kb segment on Chromosome 1. Sequence analysis of this region provides evidence for multiple independent large genomic duplications, also known as low-copy repeats, resulting in a high degree of sequence identity between *CFH* and *CFHR1–CFHR5* [9, 10]*.* The secreted protein products of these genes are related in structure, as they are composed of repetitive units (∼60 amino acids) named short consensus repeats (SCRs) [11]. In this study, we describe a novel form of nonallelic homologous recombination that results in the deletion of *CFHR1* and *CFHR3,* but leaves *CFH* intact. This deletion is associated with an increased risk of aHUS.

## Results/Discussion

Two cohorts of patients with aHUS have been studied, one from Jena, Germany and one from Newcastle, United Kingdom. For the Jena cohort of 121 aHUS patients, we used Western blotting to determine the absence of CFHR1 and CFHR3 in serum, as demonstrated for three patients in Figure 1A–1C. Complete absence of both CFHR1 and CFHR3 but presence of factor H, factor H–like protein 1, CFHR2, and CFHR4A was detected in 19 aHUS patients (16%) compared to two out of 100 control participants (χ^2^ = 10.4, *p* = 0.0012, odds ratio = 8.5). All 19 patients showed normal factor H serum levels. In three of these 19 patients, DNA analysis confirmed that the deficiency was caused by a homozygous genomic deletion. The *CFH* genes were normal, as determined by sequence analysis. Specific primers were designed which span the 113-kb region from the 3′ exons of *CFH* to *CFHR4* (Figure 2A). Failure of primers R2–R6 to amplify DNA of these patients is explained by a 84-kb deletion of a genomic fragment that includes *CFHR3* and *CFHR1* and is located downstream of *CFH* and upstream of *CFHR4*. This deletion is flanked by two duplicated segments, B and B′, which are 28,638 bp and 28,714 bp in length, respectively. B includes exons 21, 22, and 23 of *CFH* and is located 5′ of *CFHR3*. B′ includes exons 3, 4, and 5 of *CFHR-1* and is located ∼60 kb further downstream. Both segments have the same orientation, harbor several truncated long interspersed nuclear elements, and their sequence identity is >98 % [12]. The position of the deletion was mapped by amplifying regions of sequence variation between the duplicated segments. Forward and reverse primers specific for B and B′, respectively, generated a 9.2-kb product from aHUS patients' DNA, but not from control DNA. Sequence analysis allowed the identification of nucleotides from either B or B′ (Figure 2B), thus demonstrating fusion of B and B′ as a result of nonallelic homologous recombination. This was confirmed by amplification of the fused segment BB′ using primers that also generate amplification products from segment B and segment B′ of control DNA. Sequence analysis of a divergent region in B and B′ confirmed that amplification of DNA from the patient generated one product with a single sequence (Figure 2C, upper panel), while the control DNA generated two products with divergent sequences (Figure 2C, middle panel), of which one is derived from segment B and one from segment B′ (Figure 2C, lower panel). These data demonstrate fusion of segments B and B′ in the patient's DNA, and confirm that this patient is homozygous for the deletion (Figure 3A). The same results were obtained for the other patients (unpublished data). All three patients had identical breakpoints, which were mapped to a 1.9-kb region of perfect homology within or directly preceding a L1MA2 element (Figure 3B).

**Figure 1:**
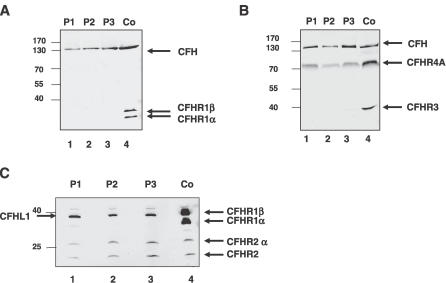
Deficiency of CFHR1 and CFHR3 in HUS Western blotting of serum from three HUS patients (lanes 1–3) and a control subject (lane 4). (A) Using a monoclonal antibody that reacts with the C terminus of CFHR1, complete deficiency of CFHR1 is seen in the three HUS patients. The normal control shows the two differently glycosylated forms of CFHR1 (CFHR1α and CFHR1β, respectively). (B) Using CFHR3 antiserum, complete deficiency of CFHR3 is seen in three HUS patients, but CFHR3 is detected in the control subject. CFHR4A is present in all samples. (C) The sera of the three HUS patients contain factor H–like protein 1 and the glycosylated and nonglycosylated forms of CFHR2 (CFHR2α and CFHR2, respectively).

**Figure 2:**
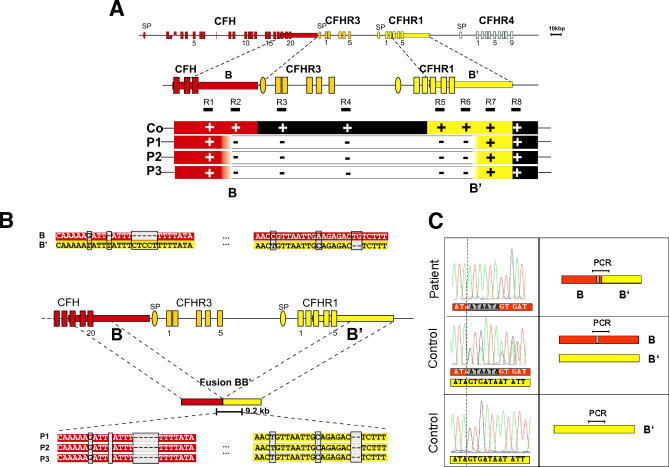
Map of the Human Factor H Gene Cluster (A) The 20 SCRs of *CFH*, the five SCRs of *CFHR3*, the fiveSCRs of *CFHR1,* and the nine SCRs of *CFHR4* are indicated by vertical bars. The position of the two duplicated homologous segments (B and B′) and that of the eight primer pairs (R1–R8) is shown. Failure (−) to amplify DNA downstream of *CFH* in the three HUS patients reveals a deletion of a genomic ∼84-kb fragment. (B) Sequence comparison of segment B (red) and segment B′ (yellow) reveals blocks with sequence diversity. Fusion of the homologous segments and localization of the breakpoints in three CFHR1/CFHR3 deficient HUS patients are indicated*.* Fusion of segments B and B′ is demonstrated by amplification of a 9.2-kb PCR fragment using a forward primer specific for segment B and a reverse primer specific for segment B′. For all three patients, sequences of block 1 are derived from segment B (red) and sequences of block 2 are derived from segment B′ (yellow). (C) Similar PCR products were generated with primers common for segment B and segment B′. Amplification of both segments is confirmed by sequence analysis. Sequence of PCR products from the patient is identical to segment B of a control DNA (upper panel). Sequence of PCR products of a control person is identical to segment B and B′ (middle panel). Identification of sequence B′ of the control person (lower panel). Sequence differences between segment B and segment B′ are indicated (grey bar).

**Figure 3:**
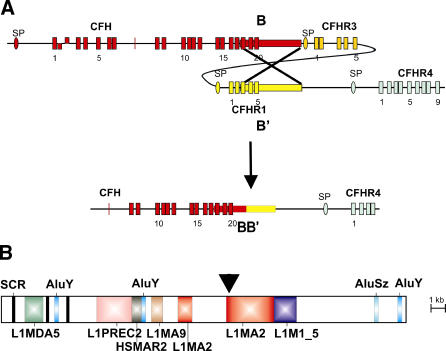
Chromosomal Rearrangement of Segments B and B′ (A) Homologous recombination between segments B and B′ in the RCA gene cluster results in deletion of the genes coding for CFHR1 and CFHR3 and fusion of segments B and B′ as described in the text. (B) The B and B′ segments in the factor H gene cluster are characterized by high sequence identity (98%) and include several truncated long interspersed nuclear elements (retrotransposons). The breakpoint identified in all three patients is indicated by the arrow.

The Newcastle cohort of 66 aHUS patients was investigated using multiplex ligation–dependent probe amplification (MLPA) [13] to measure copy number of *CFHR1* exons 2 and 3 (Table 1). The data show that deletion of *CFHR1* is strongly associated with aHUS. In the aHUS group, 28% of the patients had this deletion, compared to 6% of the control group (χ^2^ = 33.2, *p* = 1.0 × 10^−8^, odds ratio = 6.3). The following copy numbers of *CFHR1* were found in aHUS patients: zero copies, 10%; one copy, 35%; two copies, 55%. In control subjects, the copy numbers were: zero copies, 2%; one copy, 9%; and two copies 89% (χ^2^ = 28.7, *p* = 5.9 × 10^−7^). The allele frequency of *CFHR1* deletion was 30% in those patients known to carry a mutation and 27% in those without a mutation (χ^2^ = 0.16, *p* = 0.69).

**Table 1:**
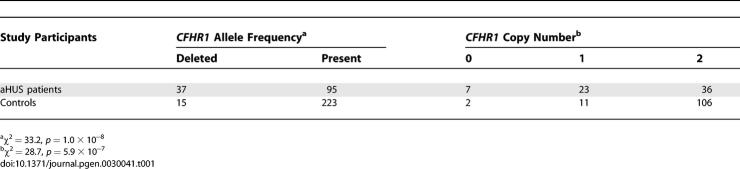
Frequency of *CFHR1* Deletion and Copy Number of *CFHR1* Exons 2 and 3 in aHUS Patients and Controls (Newcastle Cohort)

The functional effect of complete deficiency of CFHR1 and CFHR3 proteins was investigated using a modified version of a hemolytic assay with factor H–depleted plasma and sheep erythrocytes [14, 15]. Addition of heat-inactivated serum from the three patients caused increased erythrocyte lysis (Figure 4). In contrast, serum derived from a healthy individual showed dose-dependent protection. These data show that CFHR1/CFHR3–deficient plasma has reduced protective activity and suggest that the absence of CFHR1 and/or CFHR3 contributes to the defective regulation of complement activation on cell and tissue surfaces. This is underlined by the fact that these patients have normal serum levels of factor H and factor I. Similarly, we previously showed that mutant factor H derived from HUS patients displayed reduced cell binding and protection activities [16].

**Figure 4:**
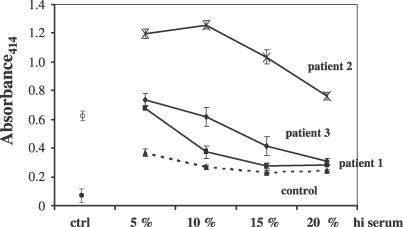
Defective Protective Activity with CFHR1/CFHR3 Deficient Plasma A novel type of hemolytic assay was used to analyze hemolytic activity of CFHR1/CFHR3 deficient human serum. Sheep erythrocytes were incubated with factor H–depleted human plasma. Sheep erythrocytes generally act as nonactivators of complement in human plasma. However, depletion of factor H from the plasma converts the sheep erythrocytes into activator surfaces (ctrl, empty circle). Addition of heat inactivated serum to depleted plasma as a source of factor H has a dose-dependent protective effect (“control”). Compared to normal human serum, sera of patient P1 to P3 with CFHR1/CFHR3 deficiency showed less protective activity as indicated by the increased lysis of erythrocytes. The classical pathway of complement activation was inhibited by the chelating agent EGTA. Erythrocytes incubated in buffer do not lyse (ctrl, filled circle). Mean values and standard deviations of three separate experiments are shown. Ctrl, control

In this study we report that hetero- and homozygous deletion of *CFHR1* and *CFHR3* through nonallelic homologous recombination events downstream of *CFH* is associated with an increased risk of aHUS. aHUS patients with deficiency of CFHR1/CFHR3 are characterized by a relatively young age (1–21 y) at disease onset. Previously, we showed that deletion of CFHR1 and CFHR3 reduces the risk of age-related macular degeneration [17]. It is fascinating that the same polymorphic variant is associated with opposite effects: an increased risk for HUS and a decreased risk for age-related macular degeneration. How could this deletion be acting? One possibility is that the absence or presence of either CFHR1 and/or CFHR3 has a disease-modifying action. Although each of the two proteins alone lacks cofactor and decay-accelerating activity, CFHR3 has a cofactor-enhancing activity [18]. In addition, both CFHR1 and CFHR3 bind C3b and heparin, thus suggesting a regulatory function in C3b processing [18].

Another possibility is that the deletion is in linkage disequilibrium with other susceptibility alleles in *CFH* or that it may affect *CFH* transcription. Understanding the functional effect of this disease-modifying deletion will help to extend our understanding of the role of complement in the pathogenesis of both aHUS and age-related macular degeneration.

## Materials and Methods

### Participants.

*Newcastle.* Sixty-six patients from Newcastle with a clinical diagnosis of aHUS were included in this study. Within this group, 15 were known to carry a *CFH* mutation, two carried a membrane cofactor protein mutation, and three carried a factor I mutation. Screening with MLPA for exons 22 and 23 of *CFH* showed that none of these patients carried a hybrid *CFH/CFHR1* gene. The allele frequency of *CFHR1* deletion in the aHUS patients was compared to 119 samples of human control DNA from a randomly selected population (healthy blood donors) obtained from the European Collection of Cell Cultures (http://www.ecacc.org.uk). The study was approved by the Northern and Yorkshire Multi-Centre Research Ethics Committee.

*Jena.* A total of 121 aHUS patients from Jena were included in this study. Of these, 19 were found to be deficient in CFHR1 and CFHR3. Genomic deletion of *CFHR1* and *CFHR3* genes was determined in three patients (Table 2). None of these three patients carried a hybrid *CFH/CFHR1* gene. The study was approved by the Research Ethics Committee of the University of Cologne, Germany, and by the Research Ethics Board of the Hospital for Sick Children, Toronto, Canada.

**Table 2:**
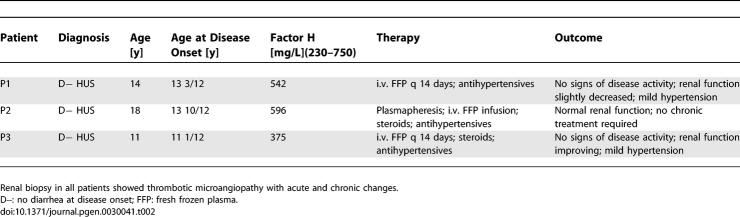
Profile of Three Patients Deficient in *CFHR1* and *CFHR3*

### Sera and Western blot analysis.

Serum samples from 121 aHUS patients (Jena cohort) and 100 anonymous healthy blood donors were assayed for the presence and mobility of factor H and CFHR1. Serum was separated by SDS-PAGE, transferred onto a membrane, and incubated with polyclonal factor H antiserum (Calbiochem-Novabiochem, http://www.emdbiosciences.com) or monoclonal C18 antibody [19], which identifies an epitope in factor H and CFHR1. CFHR3 was detected with polyclonal CFHR3 antiserum [20].

### Genetic analysis.

Genomic DNA was prepared from peripheral blood cells of three patients. Genomic DNA was amplified by PCR using specific primers R1–R8 that cover the 100-kb region downstream of the factor H gene (Table 3). Amplification of the breakpoint region and sequence analysis was performed using primers P9.2 and B1 and B2. For amplification of segment B and B′, primer P was used. The sequence of the amplified products was determined using an ABI 3100 sequence analyzer (Applied Biosystems, www.appliedbiosystems.com). The sequences were compared to that of the genomic DNA of Chromosome 1. The sequences of segment B and segment B′ were compared and analyzed for repeat content using CENSOR (http://www.girinst.org).

**Table 3:**
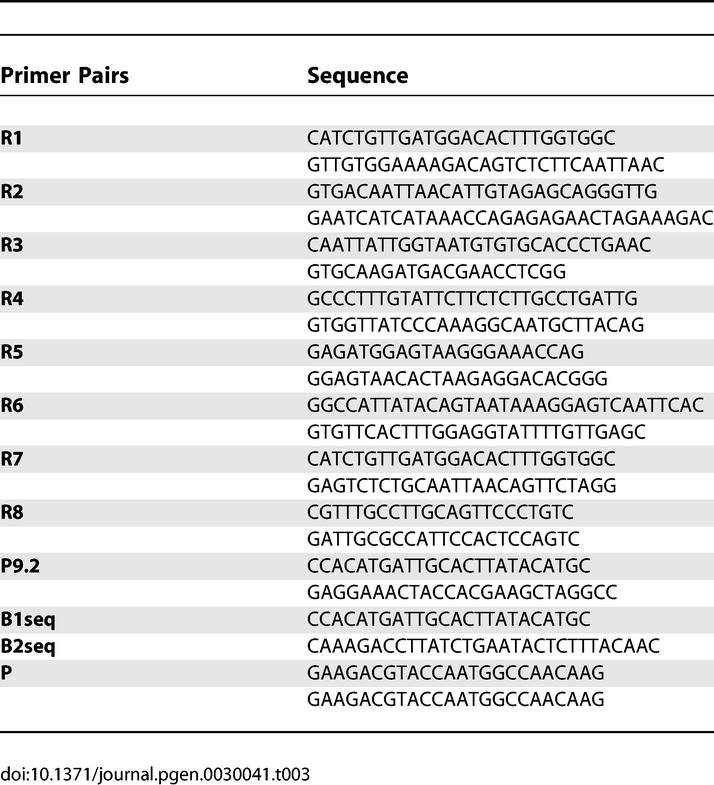
Primer Sequences for PCR Amplification and Sequencing

### Erythrocyte lysis assay.

Factor H–depleted human plasma was prepared by immunoadsorbance of factor H from normal human plasma. Polyclonal factor H antiserum (Merck Biosciences, http://www.merckbiosciences.co.uk) was covalently coupled to a 1-ml HiTrap NHS (N-hydroxy succimide) column (GE Health Care, http://www.gehealthcare.com). Factor H depletion was confirmed by Western blotting, and ELISA showed that about 66% of the protein was removed. Depleted plasma was directly used for hemolytic assays. Hemolytic experiments were performed in VBS buffer (veronal buffered saline, 144 mM NaCl, 7 mM MgCl_2_, and 10 mM EGTA, pH 7.4). Increasing amounts of heat-inactivated (10 min at 56 °C) CFHR1/CFHR3–deficient serum or control serum were added to the depleted plasma and 2 × 10^7^ sheep erythrocytes. Following incubation at 37 °C for 30 min, the mixture was cleared by centrifugation and the absorbance in the supernatants was measured at 414 nm. Using human complement active plasma sheep erythrocytes serve as non-activators of complement. These cells are protected from lysis and no hemoglobin is released. However, when factor H is depleted from human plasma, this plasma has the ability to lyse sheep erythrocytes, as demonstrated by the release of hemoglobin and an increase in absorbance. Addition of purified factor H or normal human heat-inactivated serum reconstitutes protection of erythrocytes [15].

### Multiplex ligation–dependent probe amplification.

The MLPA reaction has been previously described [13]. In this study, a completely synthetic probe set was used, obviating the need for a cloning step in the production of probes. Probes were designed to determine dosage for exons 2 and 3 of *CFHR1,* along with control probes for *MSH2* exon 1 and *MLH1* exon 19. Each probe pair hybridises to immediately adjacent targets at the sequence of interest. Hybridisation sequences are shown in Table 4. Probe pairs also contain binding sites for primers used in the MLPA reaction, as well as stuffer sequence to ensure that each amplified probe product is of a unique length. Oligonucleotides were obtained from TAG Newcastle, (http://www.vhbio.com). Righthand probes were 5′-phosphorylated and purified by desalting.

**Table 4:**
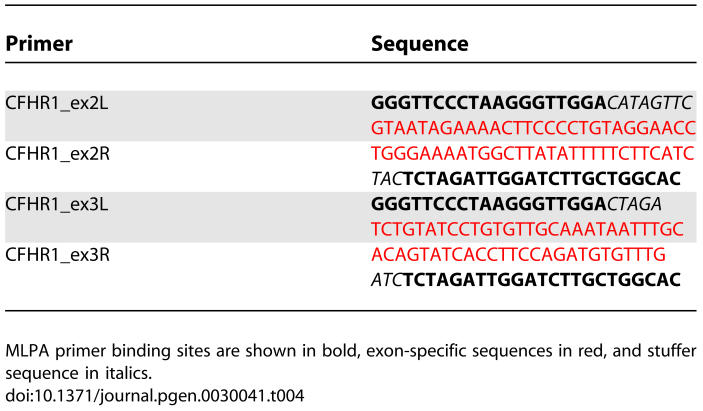
MLPA Primers

Reagents for the MLPA reaction were purchased from MRC-Holland (http://www.mrc-holland.com). The ligation reactions were carried out according to the manufacturer's recommended protocol using 100–200 ng of genomic DNA and 2 fmol of probe. Incubations and PCR amplification were carried out on a DNA Engine Tetrad 2 thermal cycler (http://www.bio-rad.com). Amplified products were diluted 10-fold to give peak heights within the quantitative range (approximately 100–4,000 units) on the ABI PRISM 3130 Genetic Analyzer capillary electrophoresis system (Applied Biosystems, http://www.appliedbiosystems.com). Diluted product (1 μl) and 0.5 μl of ROX 500 internal size standard (Applied Biosystems) were made up to 10 μl using dH_2_O and samples were run on the ABI 3130. Peak areas for each sample were determined using the proprietary Genemapper software (Applied Biosystems) and dosage quotients calculated.

## Supporting Information

### Accession Numbers

The National Center for Biotechnology Information (NCBI) (http://www.ncbi.nlm.nih.gov) accession number for the sequences discussed in this paper are Homo sapiens Chromosome 1 genomic DNA (NT_004487.18/Hs1_4644), *MLH1* (NM_000249), and *MSH2* (NM_000251).

## Abbreviations

aHUS: atypical hemolytic uremic syndrome
CFH: complement factor H
CFHR: complement factor H–related
HUS: hemolytic uremic syndrome
MLPA: multiplex ligation–dependent probe amplification
SCR: short consensus repeats

## References

[pgen-0030041-b001] Zipfel PF, Skerka C (2006). Complement dysfunction in hemolytic uremic syndrome. Curr Opin Rheumatol.

[pgen-0030041-b002] Kavanagh D, Goodship THJ, Richards A (2006). Atypical haemolytic uraemic syndrome. Br Med Bull.

[pgen-0030041-b003] Caprioli J, Castelletti F, Bucchioni S, Bettinaglio P, Bresin E (2003). Complement factor H mutations and gene polymorphisms in haemolytic uraemic syndrome: The C-257T, the A2089G and the G2881T polymorphisms are strongly associated with the disease. Hum Mol Genet.

[pgen-0030041-b004] Goicoechea de Jorge E, Harris CL, Esparza-Gordillo J, Carreras L, Arranz EA (2006). Gain-of-function mutations in complement factor B are associated with atypical hemolytic uremic syndrome. Proc Natl Acad Sci U S A.

[pgen-0030041-b005] Dragon-Durey MA, Loirat C, Cloarec S, Macher MA, Blouin J, Nivet H, Weiss L, Fridman WH, Fremeaux-Bacchi V (2005). Anti-factor H autoantibodies associated with atypical hemolytic uremic syndrome. J Am Soc Nephrol.

[pgen-0030041-b006] Lupski JR, Stankiewicz P (2005). Genomic disorders: Molecular mechanisms for rearrangements and conveyed phenotypes. PLoS Genet.

[pgen-0030041-b007] Venables JP, Strain L, Routledge D, Bourn D, Powell HM (2006). Atypical haemolytic uraemic syndrome associated with a hybrid complement gene. PLoS Med.

[pgen-0030041-b008] Heinen S, Sanchez-Corral P, Jackson MS, Strain L, Goodship JA (2006). De novo gene conversion in the RCA gene cluster (1q32) causes mutations in complement factor H associated with atypical hemolytic uremic syndrome. Hum Mutat.

[pgen-0030041-b009] Diaz-Guillen MA, Rodriguez de Cordoba S, Heine-Suner D (1999). A radiation hybrid map of complement factor H and factor H-related genes. Immunogenetics.

[pgen-0030041-b010] Zipfel PF, Jokiranta TS, Hellwage J, Koistinen V, Meri S (1999). The factor H protein family. Immunopharmacology.

[pgen-0030041-b011] Zipfel PF, Skerka C (1994). Complement factor H and related proteins: An expanding family of complement-regulatory proteins?. Immunol Today.

[pgen-0030041-b012] Male DA, Ormsby RJ, Ranganathan S, Giannakis E, Gordon DL (2000). Complement factor H: Sequence analysis of 221 kb of human genomic DNA containing the entire fH, fHR-1 and fHR-3 genes. Mol Immunol.

[pgen-0030041-b013] Schouten JP, McElgunn CJ, Waaijer R, Zwijnenburg D, Diepvens F (2002). Relative quantification of 40 nucleic acid sequences by multiplex ligation-dependent probe amplification. Nucleic Acids Res.

[pgen-0030041-b014] Pangburn MK (2002). Cutting edge: Localization of the host recognition functions of complement factor H at the carboxyl-terminal: Implications for hemolytic uremic syndrome. J Immunol.

[pgen-0030041-b015] Heinen S, Józsi M, Hartmann A, Noris M, Remuzzi G (2007). Hemolytic Uremic Syndrome: A factor H mutation (E1172Stop) causes defective complement control at the surface of endothelial cells. J Am Soc Nephrol.

[pgen-0030041-b016] Manuelian T, Hellwage J, Meri S, Caprioli J, Noris M (2003). Mutations in factor H reduce binding affinity to C3b and heparin and surface attachment to endothelial cells in hemolytic uremic syndrome. J Clin Invest.

[pgen-0030041-b017] Hughes AE, Orr N, Esfandiary H, Diaz-Torres M, Goodship T (2006). A common CFH haplotype, with deletion of *CFHR1* and *CFHR3*, is associated with lower risk of age-related macular degeneration. Nat Genet.

[pgen-0030041-b018] Hellwage J, Jokiranta TS, Koistinen V, Vaarala O, Meri S (1999). Functional properties of complement factor H-related proteins FHR-3 and FHR-4: Binding to the C3d region of C3b and differential regulation by heparin. FEBS Lett.

[pgen-0030041-b019] Oppermann M, Manuelian T, Jozsi M, Brandt E, Jokiranta TS (2006). The C-terminus of complement regulator Factor H mediates target recognition: Evidence for a compact conformation of the native protein. Clin Exp Immunol.

[pgen-0030041-b020] Skerka C, Kuhn S, Gunther K, Lingelbach K, Zipfel PF (1993). A novel short consensus repeat-containing molecule is related to human complement factor H. J Biol Chem.

